# Interplay of anemia in inborn errors of energy metabolism

**DOI:** 10.1038/s41598-026-58126-5

**Published:** 2026-06-17

**Authors:** Shambhavi Shetye, Y. Ramesh Bhat, Leslie Edward S. Lewis, Ajeetkumar Patil, Nalini Bhaskaranand, B. S. Varashree, Saritha U. Kamath

**Affiliations:** 1 Department of Medical Laboratory Technology, Manipal College of Health Professions, Manipal Academy of Higher Education, Manipal, 576104 Karnataka India; 2 Department of Paediatrics, Kasturba Medical College, Manipal Academy of Higher Education, Manipal, 576104 Karnataka India; 3 Manipal Institute of Applied Physics, Manipal Academy of Higher Education, Manipal, 576104 Karnataka India; 4 Hitech Medicare Hospital & Research Centre, NH 66, 576103 Ambalpadi, Udupi, Karnataka India; 5 Department of Biochemistry, Kasturba Medical College, Manipal Academy of Higher Education, Manipal, 576104 Karnataka India

**Keywords:** Diseases, Medical research

## Abstract

Inborn errors of energy metabolism (IEEM) are inherited conditions that typically present with impaired mitochondrial energy production and multisystem involvement, leading to adverse developmental outcomes. Iron is essential for mitochondrial function, and its deficiency may influence disease manifestations. The study aimed to evaluate the frequency of anemia and assess red blood cell indices, including the Mentzer Index (MI), in children with IEEM. This prospective observational study analyzed RBC indices in children (0-3 years) with clinically detected IEEM. Anemia was defined as per WHO criteria: hemoglobin < 11 g/dL (6-59 months). The MI was calculated and compared with age-matched controls. Anemia was observed in both the IEEM and control groups, with no significant difference between the two. Among individuals with IEEM, 78.3% had an MI ≥ 13, compared with 88.5% of controls; the difference was not statistically significant. Network analysis of complete blood count parameters demonstrated distinct clustering patterns in the IEEM group compared to controls. Although the proportion of lymphocytes and absolute lymphocyte counts were higher in the IEEM group, these differences were not statistically significant. Anemia was prevalent in both groups, with no significant difference observed between IEEM and controls. Network analysis demonstrated variations in hematological patterns; however, these findings are exploratory and do not establish underlying mechanisms. They may reflect complex interactions between metabolic and hematological factors. **CTRI Number**: CTRI/2023/11/060330.

**Supplementary Information:**

The online version contains supplementary material available at https://doi.org/10.1038/s41598-026-58126-5.

## Introduction

Iron is one of the metabolically active micronutrients, and its ability to exist in a redox state makes it important to biological processes^1^. Iron is well recognized for its significant role in hemoglobin synthesis and oxygen transport; additionally, it plays an important role in DNA synthesis, immune function, regulating oxidative stress, and cellular energy production^2^. Iron-containing proteins are integral to cellular energy production, including cytochromes, iron-sulfur cluster proteins such as mitochondrial aconitase, an important component of mitochondrial respiratory chain complexes I-III^3^. Iron is essential for overall tissue development and normal physiological functioning. Approximately two-thirds of total body iron is localized within the erythropoietic system, primarily in red blood cells. Beyond this, iron is widely distributed at the subcellular level, within the mitochondria, endoplasmic reticulum, and nucleus, being particularly rich in iron-containing and iron-binding proteins. More than 400 genes representing nearly 2% of the human genome, are involved in iron metabolism, transport, storage, and regulation, underscoring the fundamental role of iron in cellular and systemic biology^4^. The iron content within red blood cells, predominantly in the form of hemoglobin, is approximately 2,500 mg, while about 600 mg is stored as ferritin or hemosiderin^5^.

Iron deficiency represents a state in which total body iron stores are depleted, often without an immediate impact on hemoglobin levels. In contrast, iron deficiency anemia develops when iron depletion progresses to a level that compromises hemoglobin production, resulting in reduced hemoglobin level and clinically apparent anemia^6–8^. Iron deficiency is most prevalent during infancy and early childhood, representing the second most common nutritional deficiency during adolescence^9^. Global estimates indicate that approximately 40% of children aged 6-59 months are affected by anemia, with iron deficiency (50%) and thalassemia being the leading causes^10,11^. The WHO regions of South-East Asia and Africa are the most affected, with an estimated 103 million children affected in Africa and 83 million in South-East Asia^10^. A high proportion of children aged 6-59 months are affected by anemia, with the highest prevalence reported in India (53.4%), followed by Myanmar (49.6%) and Cambodia (49.0%)^12^. While iron deficiency is the most common cause of anemia, inborn errors of metabolism can lead to or may present with anemia by disrupting either mitochondrial energy pathways or iron-dependent enzymatic processes, resulting in overlapping clinical and biochemical features^13^. Iron deficiency is commonly diagnosed by measuring serum ferritin levels. However, ferritin acts as an acute-phase reactant, and its concentration may be altered in the presence of inflammation^14–16^. In addition to serum ferritin, red blood cell indices are also used for diagnosing anemia and iron deficiency, including the Kerman index, Mentzer index, Shine and Lal index, and the England and Fraser index. The Mentzer index (MI), first described by William Mentzer, is calculated by dividing the mean corpuscular volume by the red blood cell count. The diagnostic accuracy of these indices has been evaluated in multiple studies^17^. The MI demonstrated high diagnostic utility in differentiating iron deficiency anemia from thalassemia, with a sensitivity of 98.7% and a specificity of 82.3%, outperforming other hematological indices^18,19^. This index showed a positive predictive value of 79% for screening iron deficiency anemia^20^.

The present study aimed to evaluate the frequency of anemia among children aged 0-3 years with clinically detected inborn errors of energy metabolism (IEEM) disorders and to assess the utility of the MI as a screening tool for identifying probable iron deficiency anemia. In the absence of serum ferritin and other confirmatory iron status parameters, red cell indices-based calculations were used as an initial hematological assessment. This approach is intended to highlight the potential role of simple, routinely available indices in identifying iron deficiency in resource-limited settings and in populations where extensive biochemical evaluation may not be feasible.

## Materials & methods

### Study design

This study used a prospective observational study design.

### Study population

The study was conducted among pediatric participants aged 0-3 years who attended a tertiary care hospital in Karnataka, India. A total of 63 children were included in each case group and an age-matched control group. The case group included children with clinically detected IEEM who had undergone a complete baseline biochemical metabolic evaluation^21^. All participants underwent a baseline biochemical metabolic evaluation, including lactate, pyruvate, and lactate-to-pyruvate ratio as indicators of cytosolic redox status. Further targeted investigations were performed based on clinical suspicion. The IEEM group included children with clinically suspected inborn errors of energy metabolism, representing a heterogeneous spectrum of disorders affecting cellular energy pathways. This may encompass primary mitochondrial disorders as well as conditions such as organic acidemia and fatty acid oxidation defects that can exhibit secondary mitochondrial dysfunction. The control group consisted of age-matched (0-3 years) children undergoing routine clinical evaluation or baseline metabolic screening, with no clinical suspicion, family history, or biochemical evidence suggestive of metabolic disorders. Children with conditions known to significantly affect metabolic or hematological parameters were excluded from the control group. To detect a standardized effect size of 0.5 with 80% power at a 95% confidence level, a minimum of 63 children were required to be enrolled in each group.

### Diagnostic classification

This observational study focused on a population referred for metabolic evaluation of suspected inborn errors of energy metabolism. Participants in the IEEM group were included based on clinical suspicion or diagnosis of inborn errors of energy metabolism, defined by a family history of metabolic disorder, unexplained sibling death, developmental delay, or nonspecific symptoms such as poor feeding, vomiting, lethargy, or failure to thrive. Then, comprehensive clinical evaluations and relevant laboratory assessments are performed to identify potential metabolic disorders. Diagnosis was established through a combination of:

1. Clinical evaluation by the physician based on symptoms suggestive of IEEM.
2. Routine hematological and biochemical tests, including lactate, pyruvate, and lactate-to-pyruvate ratio.
3. Further metabolic investigations, such as amino acid analysis, urine organic acid analysis, and GC-MS, were performed as needed for confirmatory purposes.
4. Genetic confirmation was considered wherever available through molecular testing.

The clinical assessment was followed by laboratory tests, as outlined in Table 1.

**Table 1:** Classification of IEEM cases included in the Study.

Sr. No	Diagnsotic category	Criteria used for diagnosis	Number of individuals diagnosed
1	Biochemically supported IEEM	Abnormal lactate, pyruvate, L/P ratio, ammonia, and glucose.	50
2	Metabolically confirmed IEEM	Supported by amino acid analysis, urine organic acids, biopsy, TMS, and GC-MS.	7
3	Genetically confirmed IEEM	Confirmed by mutation analysis or molecular testing.	6
	Total IEEM cases included		63

Since this was an observational study, not all patients underwent complete genetic confirmation; therefore, cases were categorized based on the highest level of diagnostic certainty available. Clinical and laboratory data were systematically extracted from the patients’ medical records.

The complete blood counts of all participants were extracted and evaluated, and the MI was calculated from the red cell indices, which were then subsequently compared between the groups. The Mentzer Index was used as a screening test to indicate possible iron deficiency; no confirmatory iron studies, such as serum ferritin or transferrin saturation, were available. All methods were carried out in accordance with relevant guidelines and regulations and in compliance with the principles of the Declaration of Helsinki. The study was approved by the Institutional Ethics Committee of Kasturba Medical College and Kasturba Hospital (Approval No.: 184/2023, Date: 08 November 2023). Before inclusion in the study, written informed consent was obtained from the parents of all participants. The study was prospectively registered with the Clinical Trials Registry-India (CTRI) under registration number CTRI/2023/11/060330 (Date: 29/11/2023).

###  Data analysis

Demographic details of all participants were recorded. The MI was calculated by dividing the mean corpuscular volume (MCV) in femtoliters (fL) by the red blood cell (RBC) count in millions per microliter. The calculated Mentzer index values were used for further comparative analysis between the study groups^22^. Statistical analysis was performed using R (version 4.4.1), RStudio (2025.09.1 + 401), and Jamovi (version 2.4.11). Normality was assessed using the Shapiro-Wilk test. Parametric or non-parametric tests were applied based on data distribution. Categorical variables were expressed as frequencies and percentages and compared using Fisher’s exact test. A p-value < 0.05 was considered statistically significant. Network analysis was performed using R software.

## Result

A total of 126 children were included in the study, comprising 63 IEEM and 63 controls. Data were unavailable for six children in the IEEM and control group; therefore, these participants were excluded from further analysis. Male children predominated in both groups, accounting for 65.1% of IEEM and 58.7% of controls; however, the gender distribution was comparable. Controls were matched for age and sex; no additional matching for other clinical or biochemical variables was performed, which should be considered when interpreting the findings. Detailed demographic and characteristics of all the participants are given in Table 2.

**Table 2:** Baseline demographic and hematological characteristics of IEEM and controls.

Characteristics		IEEM group (Mean ± SD)	Control (Mean ± SD)
Total number of participants (n)		60	60
Age in months		14.1 ± 12.8	13.3 ± 10.3
0–6 months (n)		24	17
6–12 months (n)		16	27
12–24 months (n)		11	11
24–36 months (n)		9	5
Hb Reference level: 11.1–14.2 g/dl		11.1 ± 1.89	10.7 ± 2.26
Hct Median (IQR) Reference level: 30–38%		33.0 (4.85)	32.1(6.9)
MCV Reference level: 72–84 fl.		77.4 ± 12.2	75.3 ± 12.4
RBC Reference level: 3.9–5.1 10^6^/ul		4.4 ± 0.829	4.3 ± 0.640
MCH Reference level: 25–29 pg		25.7 ± 4.61	25.1 ± 4.81
MCHC Reference level: 32–36 g/dl		33.1 ± 1.52	33 ± 2.13
RDW Reference level: 11.6–14%		15.9 ± 2.06	16.9 ± 4.03
Anaemia	Anaemia	10.3%	16.7%
	No anaemia	39.7%	33.3%
MI category	≥ 13	47 (78.3)	54 (88.5)
	< 13	13 (21.7)	7 (11.5)

**Fig. 1:**
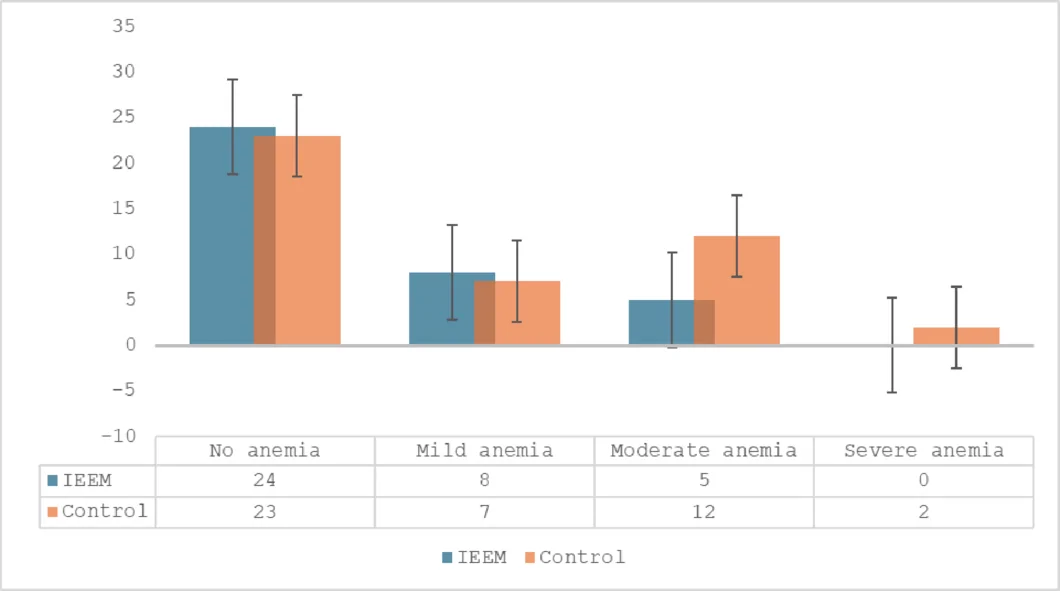
Pattern of anemia in the study cohort.

The mean hemoglobin concentration was 11.1 ± 1.89 g/dl for IEEM and 10.7 ± 2.26 g/dl for controls. It was lower in the control group than in the IEEM group. Independent samples t-test analysis showed no statistically significant differences between the IEEM and control groups. WHO criteria were used to classify anemia severity based on hemoglobin concentration (g/dl)^23^. Using the World Health Organization (WHO) criteria, anemia was assessed in both groups and categorized by severity; the distribution is summarized in Table 3. The results are shown in Fig. 1.

**Table 3:** Anemia frequency in the IEEM and control of age 6 months to 36 months based on the WHO criteria.

Severity of anemia	Age groups (Months)	Group	Count (%)
No anemia	6–23	IEEM	11 (13.60%)
		Control	14 (17.30%)
	24–59	IEEM	13 (16.00%)
		Control	9 (11.10%)
Mild	6–23	IEEM	3 (3.7%)
		Control	6 (7.4%)
	24–59	IEEM	5 (6.2%)
		Control	1 (1.20%)
Moderate	6–23	IEEM	3 (3.7%)
		Control	7 (8.6%)
	24–59	IEEM	2 (2.5%)
		Control	5 (6.2%)
Severe	6–23	IEEM	0
		Control	2 (2.5%)
	24–59	IEEM	0
		Control	0

Due to the smaller cell counts, Fisher’s exact test was used to compare the severity of anemia between the IEEM and control groups. No statistically significant association was observed between anemia severity and study group. Further analysis using a binary classification of anemia also showed no significant difference in anemia prevalence between cases and controls (*p* = 0.795; OR 0.82, 95% CI 0.26–2.61). WHO reference cut-off values for anemia are defined only for children aged ≥ 6 months. Therefore, infants younger than 6 months (40% in the IEEM group and 28.3% in the control group) were excluded from the anemia analysis, as anemia cannot be reliably assessed in this age group. (Fig. 1).

**Fig. 2:**
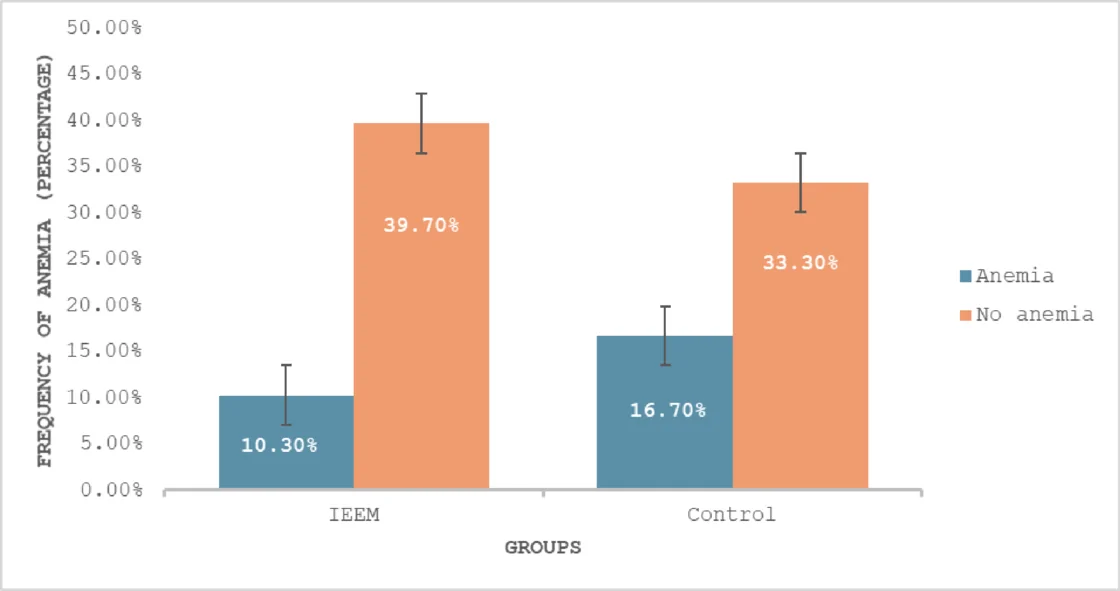
Frequency of anemia in the IEEM and control groups.

The total frequency of anemia differed between the IEEM and control groups. This distribution of anemic and non-anemic children across both groups is illustrated using a bar plot for visual comparison. (Fig. 2). The anemia frequency was higher in the control group than in the IEEM group.

### Calculation of the mentzer index (MI)

The MI was calculated as the mean corpuscular volume (MCV) divided by the red blood cell (RBC) count, and the frequency of values ≥ 13 (suggestive of possible iron deficiency) and < 13 was documented. It is depicted in Table 4.

**Table 4:** Distribution of MI in the case and control groups.

Group	MI ≥ 13	MI < 13
IEEM	47 (78.33%)	13 (21.66%)
Control	54 (88.5%)	7 (11.4%)

**Table 5:** Relationship between MI categories and anemia status.

Mentzer Index Category	Anemia	No anemia
MI ≥ 13	12	13
MI < 13	4	5

**Fig. 3:**
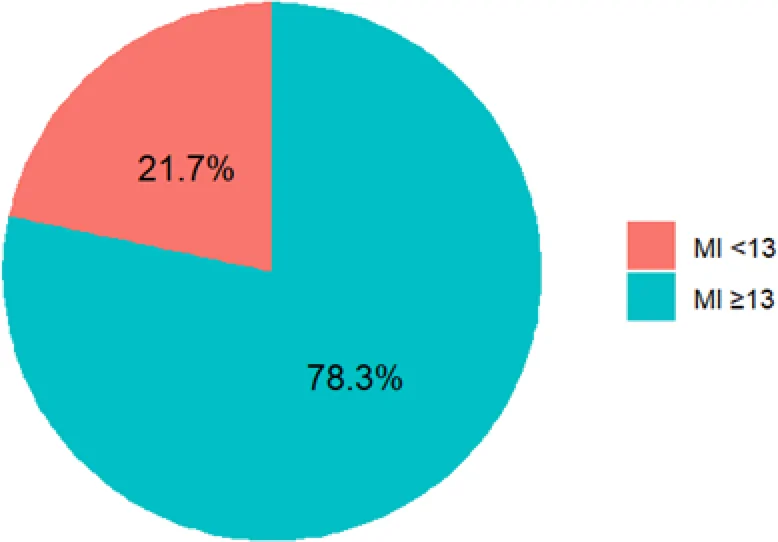
Mentzer index category in the IEEM.

**Fig. 4:**
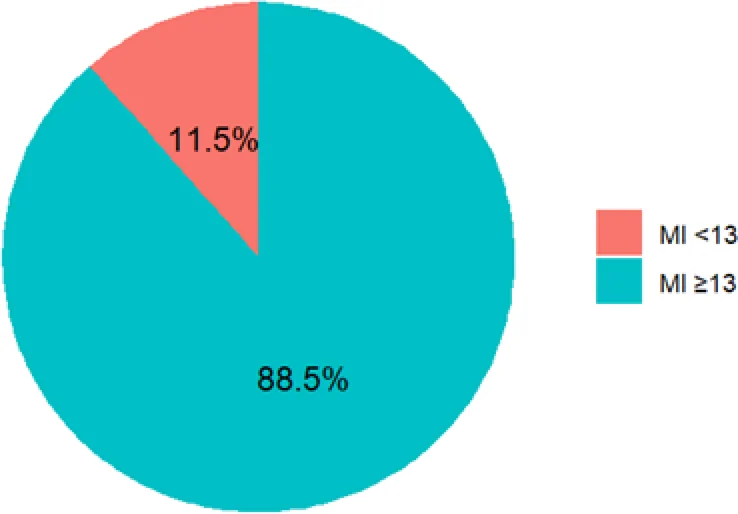
Mentzer index category in the control group.

MI categorization revealed that the majority of children in both groups had values ≥ 13, a suggestive indicator of possible iron deficiency. Among the cases, 78.3% had an MI ≥ 13, compared with 88.5% of the controls. The difference was not statistically significant. The association between the MI category and anemia status was analyzed to evaluate its clinical relevance. No significant association was observed between the MI category and anemia among cases (Fisher’s exact test, *p* = 1.00). (Table 5).

Differences in the MI were evaluated between the IEEM and control groups. The distribution of MI categories did not differ significantly between IEEM and controls (Fisher’s exact test, *p* = 0.149), indicating that an iron deficiency-suggestive pattern was not overrepresented among children with IEEM. (Figures 3 and 4)

### Network analysis

**Fig. 5:**
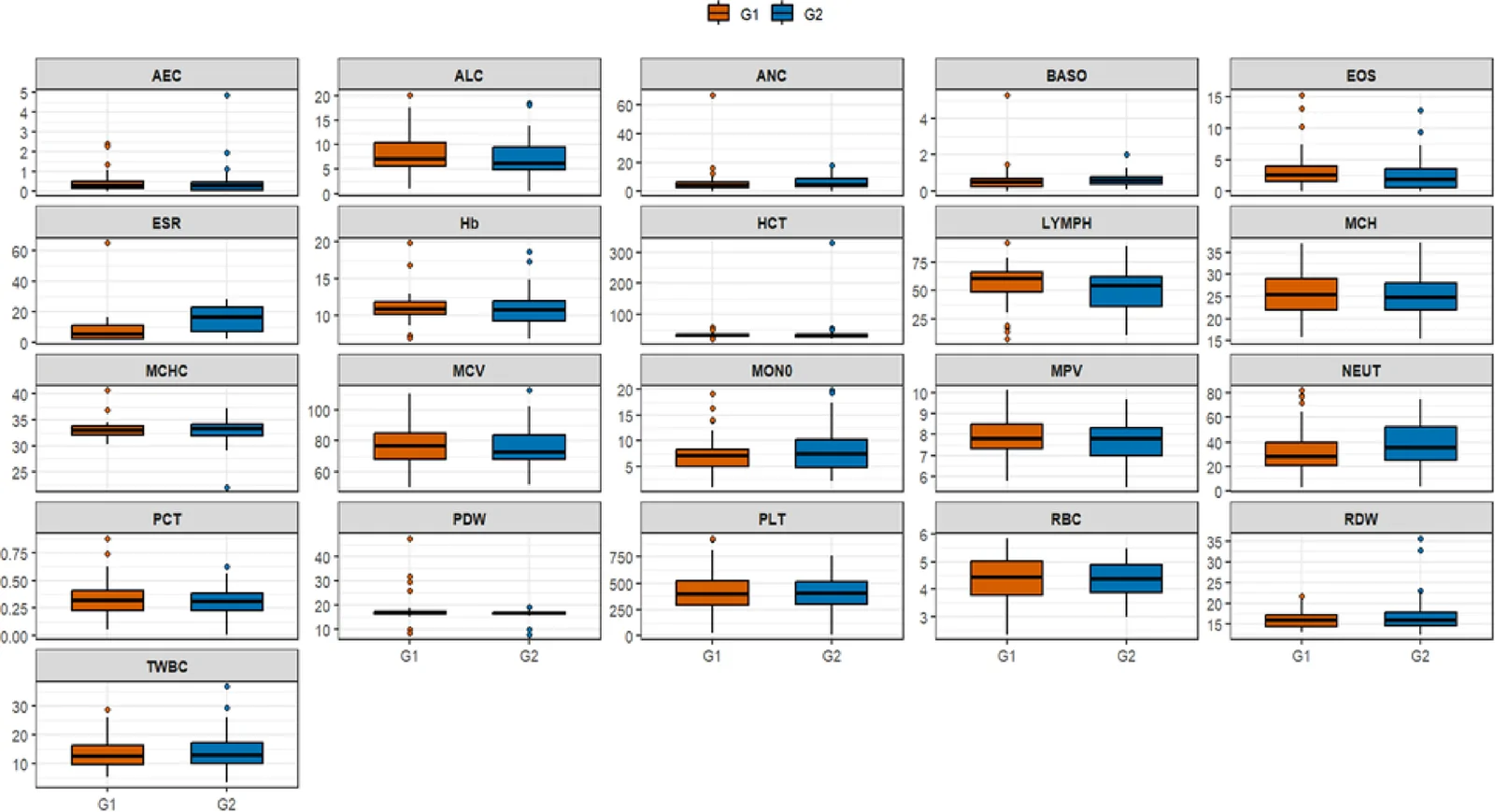
Distribution of hematological parameters across the study group.

Although conventional statistical comparisons did not reveal significant differences in individual hematological parameters between the IEEM and control groups, such univariate analyses assess each parameter in isolation. Hematological parameters are biologically interdependent and reflect coordinated physiological processes. Therefore, the absence of individual parameter differences does not rule out altered systemic interactions. Box plots of hematological parameters showed substantial overlap in median values and interquartile ranges between the IEEM (G1) and control (G2) groups for most parameters, indicating broadly comparable distribution patterns. (Fig. 5). However, this distribution-based visualization assesses parameters individually and does not capture potential alterations in the relationships among hematological variables.

**Fig. 6:**
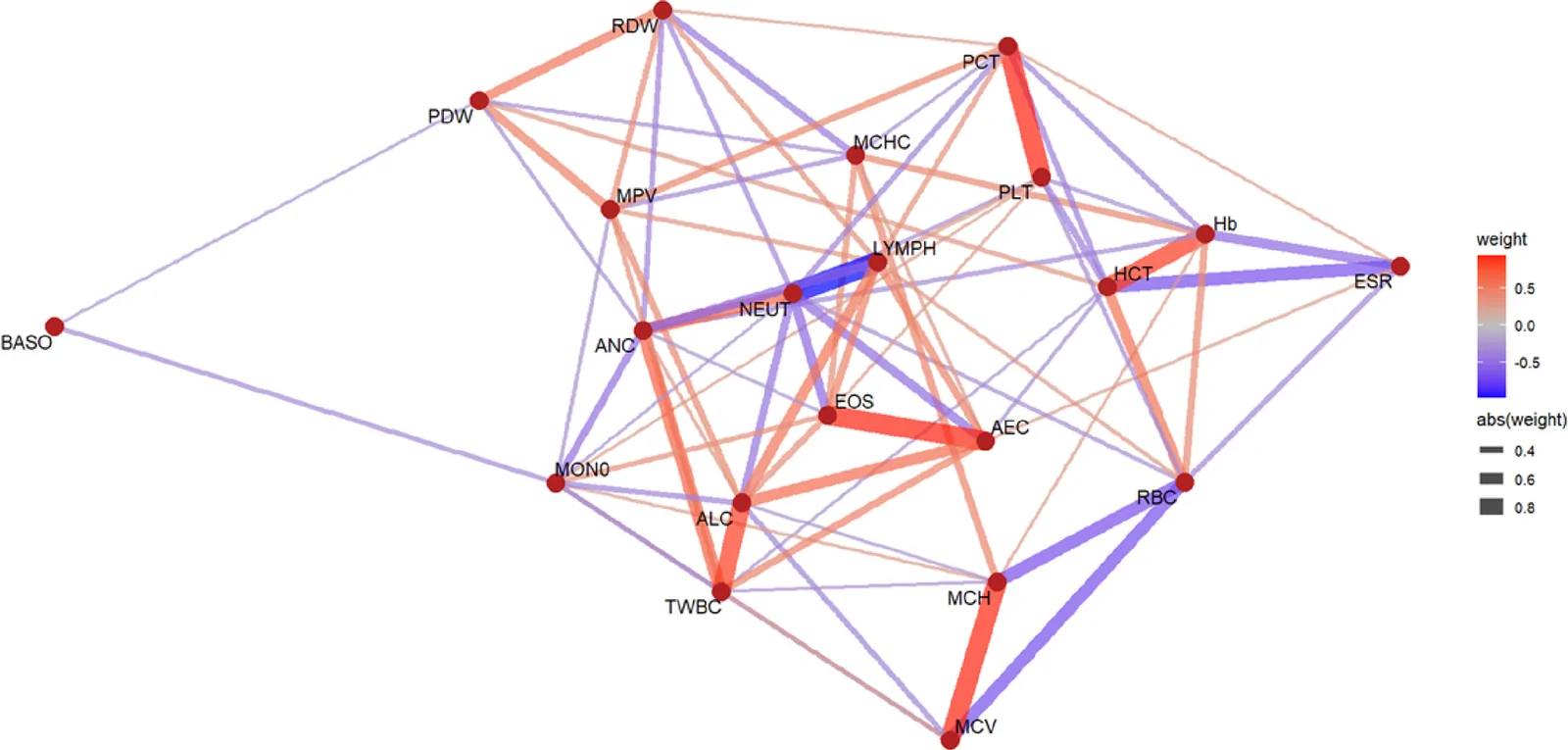
Network analysis of complete blood count parameters in the overall.

To explore potential differences in the interrelationships among hematological parameters that may not be captured by conventional statistical testing, a correlation-based network analysis was performed separately for the IEEM and control groups. Spearman correlation coefficients were calculated to account for non-normal data distribution. In earlier analyses, the focus was limited to red blood cell indices due to their established relevance in detecting and characterizing anemia. In the present analysis, however, all available hematological parameters were included to enable a comprehensive assessment of overall hematological patterns and inter-parameter relationships. First, we visualized the overall distribution patterns of all hematological parameters collectively to assess their general behavior across the study population. (Fig. 6)

Significant correlations and interconnections were observed among hematological parameters in the overall network. In the network figure, dots represent hematological parameters, and edges connect two parameters based on their interrelationships, which may be correlations or associations. Red edges indicate positive correlations, while blue or purple edges indicate negative correlations. The thickness of the edge reflects the strength of the correlation, with thicker lines representing stronger associations.

To examine whether these interaction patterns differed by disease status, correlation networks were subsequently constructed separately for the IEEM and the control group.

**Fig. 7:**
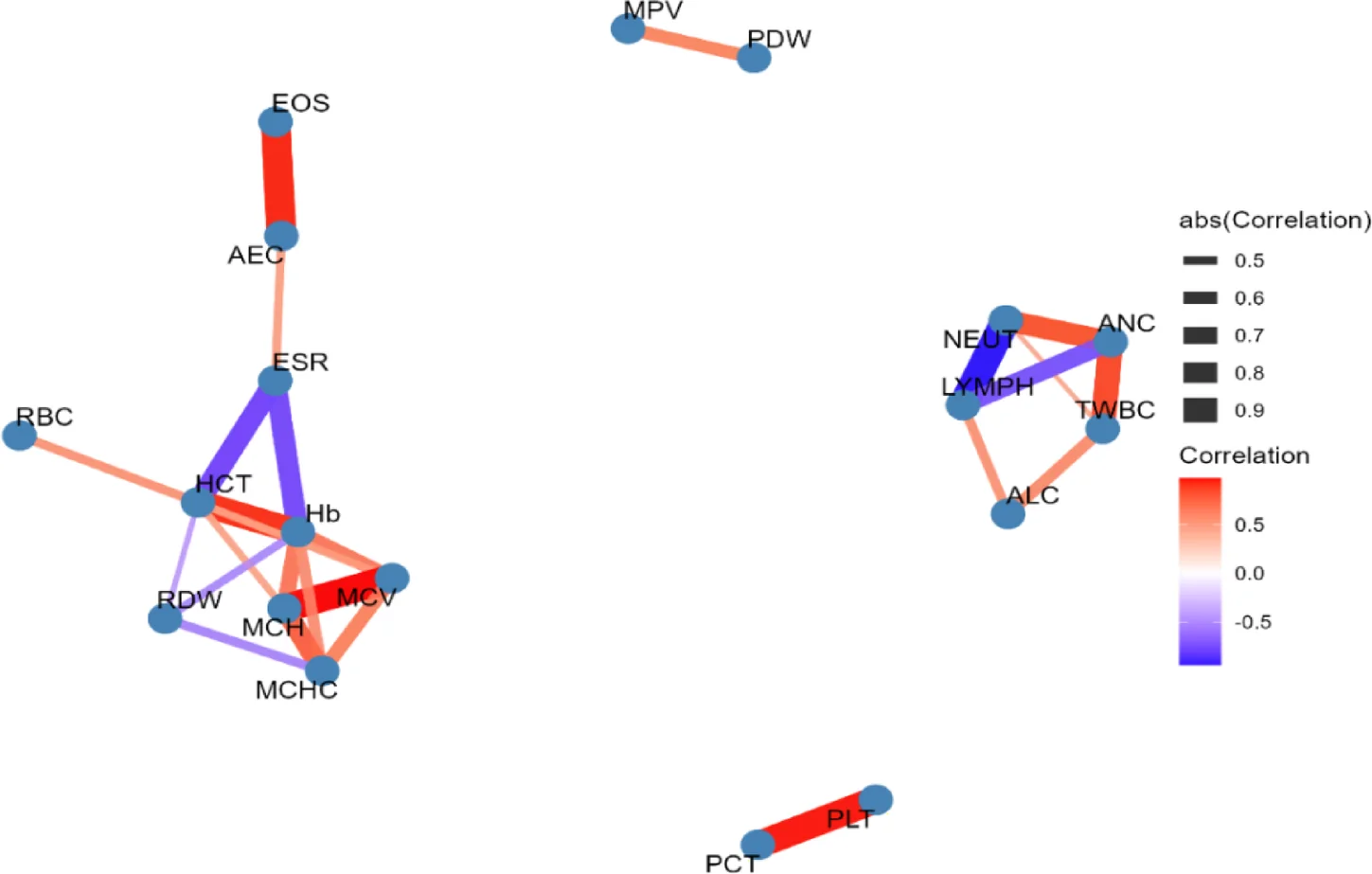
Network analysis of the control group.

In the control group, the network exhibited a well-defined, orderly structure. Red cell indices formed a compact cluster, characterized by predominantly moderate-to-strong positive correlations. Immune cell parameters formed a separate cluster, showing strong internal associations but minimal connections with erythroid indices. Platelet-related parameters (PLT, PCT) also formed an independent module, remaining largely isolated from both erythroid and immune clusters. Overall, the control network demonstrated high intra-cluster connectivity and limited inter-cluster links. (Fig. 7)

**Fig. 8:**
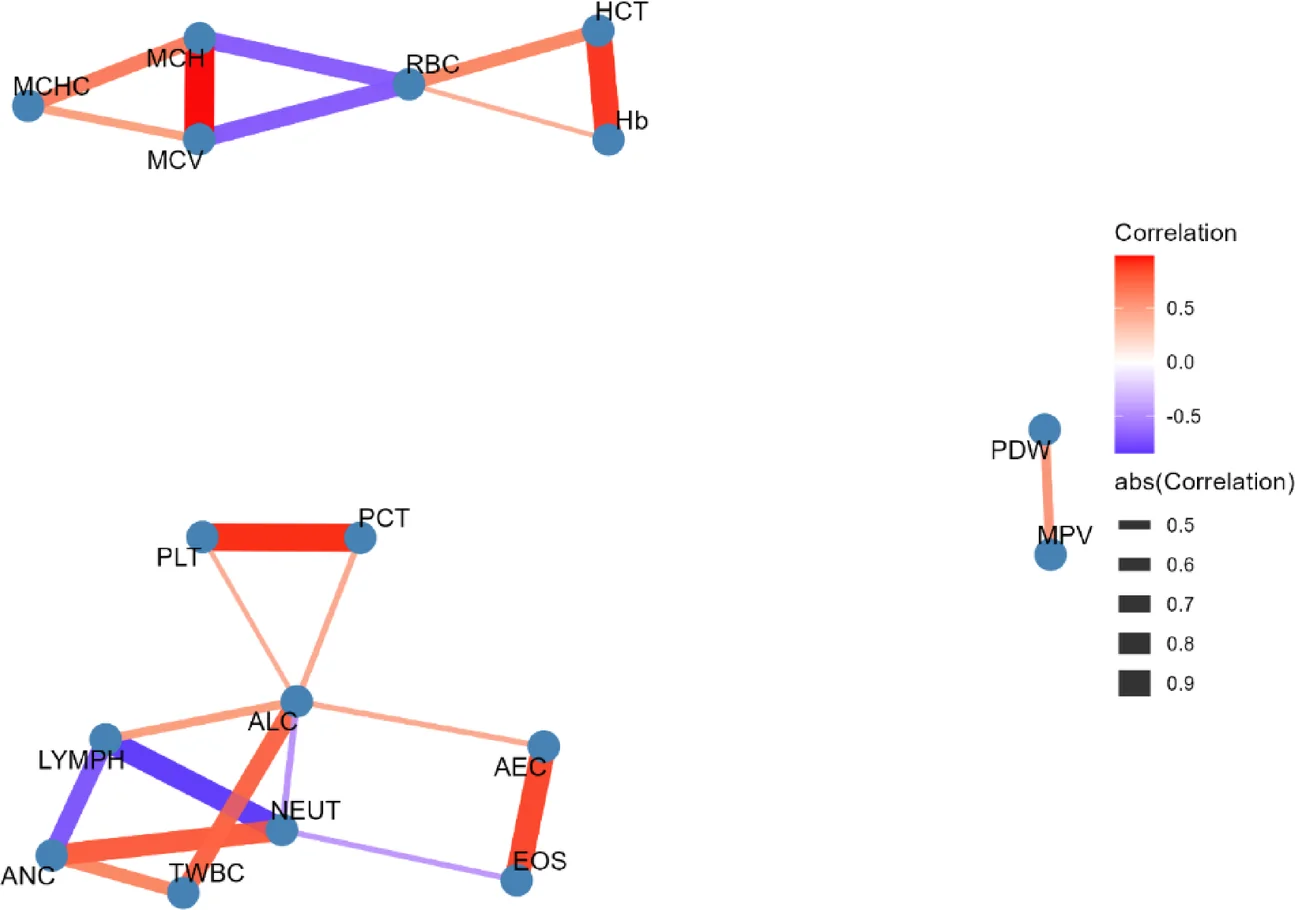
Network analysis of the IEEM group.

In the IEEM group, the network revealed numerous interrelationships, which were organized into three distinct clusters. One cluster comprised the RBC indices, in which strong negative correlations were observed between RBC and MCH and between RBC and MCV. Strong positive correlations (red edges > 0.7) between Hb, MCV, HCT, and RBC indicate a coordinated erythropoietic response. Moderate associations with platelets (PLT) and negative correlations with PDW likely reflect marrow competition during periods of rapid red blood cell turnover in infants. There were more connections between erythroid, immune, and platelet parameters, indicating increased cross-lineage interaction. This contrasts with the control group, where erythroid indices showed tight clustering with minimal external links. (Fig. 8)

## Discussion

The present study evaluated the frequency of anemia among children aged 0-3 years with clinically detected inborn errors of energy metabolism, assessed the utility of the MI as an indicator of possible iron deficiency in this population, and compared it with age- and gender-matched control groups.

Iron is essential for the mitochondria, the energy-generating units of the cell. Mitochondria stand out for their role in the biosynthesis and utilization of iron cofactors. The formation of heme and Fe-S clusters, a part of which occurs in the mitochondria^24^. The abundance of iron cofactor within the proteome of mitochondria is 3.5-fold higher than the total proteome^25^. These cofactors are integral to the electron transport chain, participate in mitochondrial anabolic and catabolic pathways, and are involved in iron storage by virtue of mitochondrial ferritin (FTMT)^26^. Multiple iron-dependent mitochondrial pathways exist in the body, as evidenced by the essential role of iron-sulfur (Fe-S) clusters in the respiratory chain, the tricarboxylic acid (TCA) cycle, mitochondrial glutathione balance, heme biosynthesis, and mitochondrial translation^27^. Thus, iron plays a major role in overall energy metabolism, which is already compromised in inborn errors of energy metabolism. However, there is limited evidence and understanding regarding the impact of iron deficiency and anemia on mitochondrial function, as well as the associated metabolic and biochemical adaptations that occur in the context of iron deficiency during energy metabolism disorders.

**Fig. 9:**
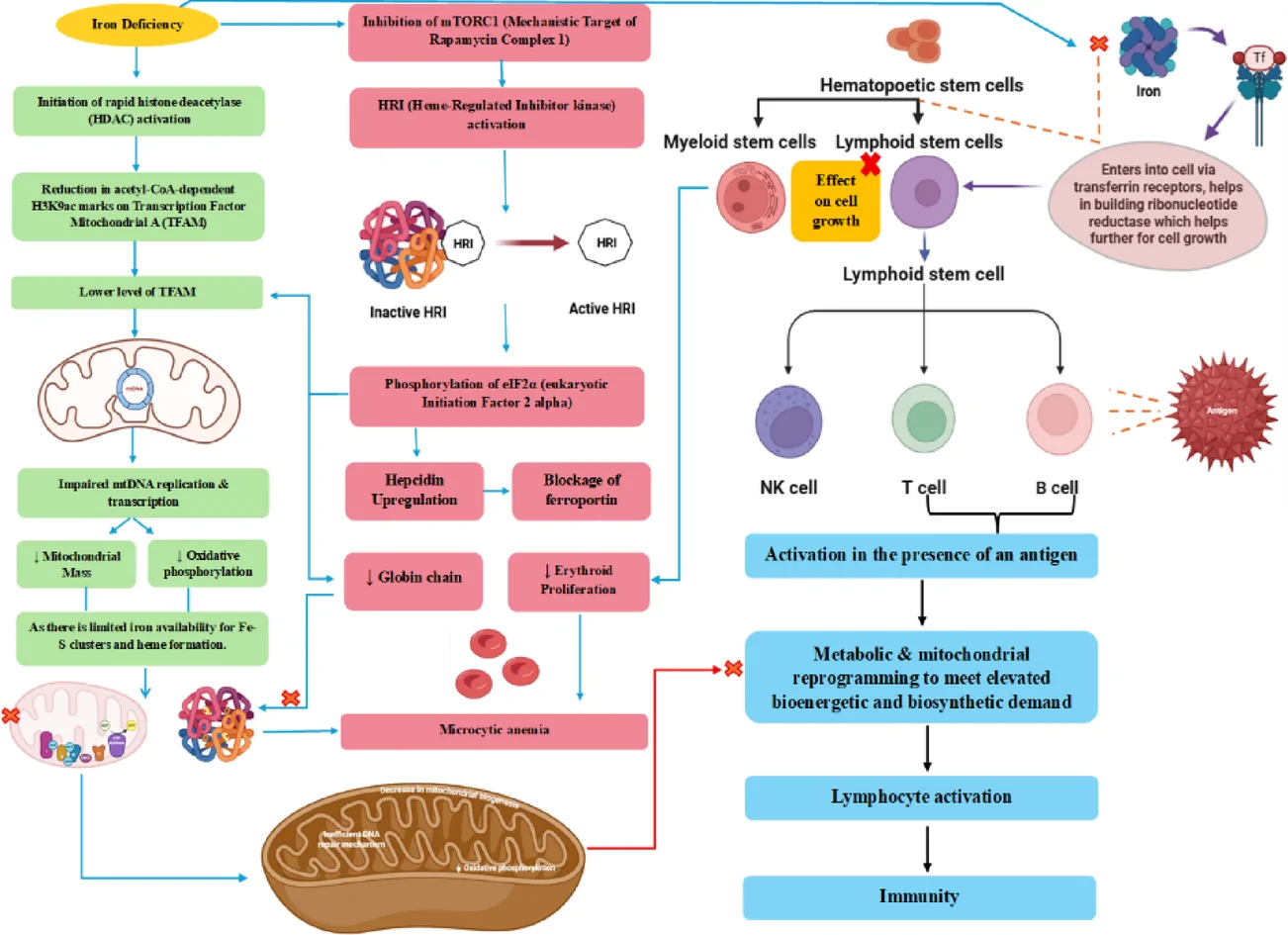
Schematic representation of the interplay between iron metabolism, mitochondrial function, erythropoiesis, and immune regulation, based on published evidence and supported by findings from the present study. The figure represents a conceptual synthesis based on findings from the current study and published literature and is intended for illustrative purposes only. Created by the authors using BioRender (https://www.biorender.com).

Iron-containing tetrapyrroles, such as heme, perform various functions within the body, including oxygen binding, electron transport, and enzymatic catalysis^28–30^. Depletion of heme may arise from environmental or genetic factors, among which iron deficiency is a major cause^31^. The reference laboratory method for evaluating and diagnosing iron deficiency is to measure serum ferritin levels^32^. Ferritin levels are typically assessed alongside additional biochemical markers, including serum iron, transferrin, transferrin saturation, and soluble transferrin receptors. This approach is emphasized in systematic reviews and aligns with established global guidelines for diagnosing iron deficiency^33^. An evaluation of 29 guidelines published by professional associations worldwide demonstrates substantial heterogeneity in the diagnostic criteria for iron deficiency. Given that iron homeostasis is tightly regulated through multiple mechanisms, iron deficiency is evidenced through a variety of biochemical and haematological indicators. Assessment of iron status across different physiological compartments, including iron storage, transport, and functional iron, can provide a comprehensive understanding of overall iron status^34^. Iron stores are typically assessed using bone marrow iron staining and serum ferritin, while iron transport is evaluated through measures such as transferrin saturation, erythrocyte protoporphyrin, and soluble transferrin receptor levels. The functional availability of iron is reflected by hemoglobin and hematocrit values. Furthermore, mean corpuscular volume (MCV) and red cell distribution width (RDW) are also helpful in classifying different types of anemia^35^. Alongside these measures, several red cell-based indices, including the MI, are commonly used to screen for anemia and to distinguish iron deficiency from other causes^36^. In settings where sample volume is limited or where practical constraints limit the performance of multiple tests, the MI offers a simple, economical screening approach. Previous studies have evaluated the diagnostic performance of the Mentzer Index. Notably, Ahmed et al. reported high sensitivity (95.24%) and specificity (93.10%) for its use in identifying iron deficiency anemia^37^. Similarly, several studies have evaluated the usefulness of MI in screening for IDA and Thalassemia trait^36,38^. This study used the MI to assess a possible iron deficiency in children with IEEM. Anemia status was also evaluated by assessing functional iron indicators, including hemoglobin, mean corpuscular volume, red cell distribution width, mean corpuscular hemoglobin, and mean corpuscular hemoglobin concentration. Although a high proportion of participants showed Mentzer Index values suggestive of possible iron deficiency (78.3% among cases and 88.5% among controls), no significant association was observed between MI categories and anemia status or functional iron indicators. Furthermore, the distribution of MI categories did not differ significantly between cases and controls. This observation contrasts with a previous study in which anemia was frequently reported in both syndromic and non-syndromic mitochondrial disorders^39^. A case report demonstrated that mutations in SLC25A38, a nuclear DNA-encoded gene encoding a mitochondrial inner membrane carrier protein, resulted in microcytic hypochromic sideroblastic anemia^40^. Anemia has been reported as a clinical feature in several mitochondrial disorders, including Pearson syndrome, Kearns-Sayre syndrome, MLASA, XLSA-A, and Leigh syndrome, and it has also been described as one of the causes of death in Pearson syndrome^41–47^. In this context, network analysis was employed to explore whether hematological parameters exhibit distinct interaction patterns in children with IEEM compared to controls, revealing clear differences in their distribution and inter-relationships. Network analysis demonstrated distinct patterns in the distribution of hematological parameters between the IEEM and control groups. For this evaluation, all complete blood count parameters, including white blood cell subtypes and platelet counts, were included. The analysis revealed group-specific clustering, with hematological parameters in the IEEM group forming distinct clusters compared to controls, indicating differences in inter-parameter relationships. In the individual parameter assessment, the proportion of lymphocytes was higher in the IEEM group (55.06 ± 17.86%) than in the control group (50.59 ± 17.26%). Similarly, the absolute lymphocyte count (ALC) was elevated in the IEEM group compared with controls, suggesting a relative lymphocyte predominance in children with IEEM. Mitochondria play a central role in immune function, particularly in lymphocytes, where they support cellular activation, metabolic remodelling, energy production, and differentiation. Previous studies have reported elevated lymphocyte proportions in patients with mitochondrial dysfunction, suggesting that mitochondrial dysfunction may contribute to immune dysregulation and altered lymphocyte homeostasis^48–50^. Mitochondrial oxidative phosphorylation is essential for meeting the energy demands of naive lymphocytes, whereas metabolic shifts toward glycolysis and glutamine oxidation support their differentiation into effector subsets during immune activation^51^. In inborn errors of metabolism involving mitochondrial dysfunction, disruption of these metabolic pathways may contribute to lymphocytosis, consistent with previously reported immune alterations in primary mitochondrial disorders. These observations highlight the need for further studies exploring mitochondrial-immune interactions in pediatric IEEM cohorts (Fig. 9). This schematic representation is based on established mechanisms reported in previous studies and is integrated with observations from the present study. The figure represents a theoretical framework derived from current literature rather than direct experimental validation. Further targeted biochemical and mechanistic studies are required to elucidate and confirm the precise pathways involved.

Inborn errors of energy metabolism in early childhood are characterized by disturbances in cellular energy metabolism, which may affect physiological processes during early development. Iron and heme are essential for mitochondrial function through their roles in iron-sulfur cluster formation, respiratory chain complexes, and enzymes of the tricarboxylic acid cycle. Alterations in iron availability have been associated with changes in mitochondrial function, including effects on heme-dependent processes and oxidative balance, as reported in previous studies. However, the mechanisms linking iron homeostasis, mitochondrial dysfunction, and erythropoiesis in IEEM remain incompletely understood. Further investigations are required to clarify the relationship between IEEM and hemoglobin synthesis, hemoglobin concentration, oxygen-carrying capacity, and associated hematological features of red blood cells, including potential structural and functional changes. In this study, we evaluated the prevalence of anemia and assessed the utility of the MI in children with Inborn errors of energy metabolism; however, no statistically significant discriminatory results were obtained. In the present study, confirmatory iron status testing (serum ferritin, serum iron, and total iron-binding capacity) was performed only in a subset of participants and not uniformly across the cohort, representing a limitation. A completely healthy pediatric control group could not be included in the study. Recruitment of healthy children poses ethical and practical challenges, as invasive procedures such as blood sampling cannot be justified in asymptomatic pediatric populations. Consequently, the control group comprised age- and sex-matched children undergoing clinical evaluation, which may limit direct comparison with an entirely healthy population. Contrary to our initial hypothesis, the frequency of anemia was not significantly higher in children with IEEM compared to controls. This finding suggests that broader nutritional and environmental factors may influence anemia in this population. However, despite similar prevalence, the observed differences in hematological network patterns indicate that the underlying biological context of anemia may differ in IEEM.

The present study and analysis focus specifically on hematological manifestations in inborn errors of energy metabolism; detailed clinical and baseline metabolic workup has been reported separately. The absence of detailed mitochondrial genetic analyses limits the interpretation of the mechanisms underlying erythropoiesis and iron handling. In addition, the lack of longitudinal nutritional and micronutrient data restricts the assessment of the role of early-life iron status. Further studies, including broader hematological evaluation, relevant genetic analyses, and longitudinal nutritional data, are needed to better understand the relationship between mitochondrial dysfunction, iron metabolism, and hematological changes in this population. The study aimed to assess a cost-effective method for screening for iron deficiency and anemia. While anemia was a focus of the evaluation, it’s important to acknowledge that there are various other underlying causes as well. Megaloblastic anemia, particularly when thiamine-dependent mechanisms are involved, can significantly impact energy metabolism by altering mitochondrial function and cellular carbon flow. Besides iron, various micronutrients, including vitamins and minerals, are crucial for sustaining mitochondrial health and supporting erythropoiesis. Notably, vitamins such as thiamine, riboflavin, folic acid, and cobalamin are vital for energy metabolism and the development of anemia. These factors were not specifically examined in the current study and warrant further exploration in future research.

## Conclusion

Anemia is common in early childhood, a period of rapid physiological growth that involves the brain and other organ systems and requires adequate nutritional support. In this context, the present study evaluated hematological markers of anemia in children with clinically diagnosed inborn errors of energy metabolism disorders, in whom baseline energy deficits may be further exacerbated by iron deficiency. The frequency of anemia did not differ significantly between the IEEM and control groups. Distinct hematological interaction patterns were observed; however, these findings are exploratory and do not establish underlying regulatory mechanisms. They may reflect the complex interplay between metabolic and hematological factors in IEEM beyond simple prevalence measures. As the analysis was limited to heme-related parameters, the findings highlight the need for confirmatory iron studies, such as serum ferritin and total iron-binding capacity, along with assessment of other micronutrients, including vitamin B1, B2, B12, B9, etc., to allow a more comprehensive evaluation of mitochondrial function in this vulnerable population and to inform targeted therapeutic strategies. Secondary analysis identified peripheral lymphocyte elevation in a subset of children with IEEM. Clinically, many children with inborn errors of metabolism present during periods of intercurrent illness, such as prolonged fever or respiratory infections, and the metabolic stress is known to exacerbate disease manifestations. During these episodes, underlying mitochondrial dysfunction may limit the ability to meet the increased energy demands of the immune response, potentially contributing to altered lymphocyte profiles. Whether lymphocyte elevation reflects acute infection, metabolic decompensation, or their combined effect remains uncertain and needs further focused studies in pediatric IEM cohorts.

## Electronic Supplementary Material

Below is the link to the electronic supplementary material.

## Data Availability

The data that support the findings of this study are available on request from the corresponding author. The data are not publicly available due to privacy or ethical restrictions.
