# Supplementary material for: Interplay of anemia in inborn errors of energy metabolism
Source: Sci Rep. 2026 Jun 17;16:27643. doi: 10.1038/s41598-026-58126-5 (PMC13538414; doi:10.1038/s41598-026-58126-5)
Supplement: Supplementary file 1 [file 41598_2026_58126_MOESM1_ESM.docx]

**Supplementary file:**

1. Study Protocol
2. Strobe checklist
